# Subwavelength grating enabled on-chip ultra-compact optical true time delay line

**DOI:** 10.1038/srep30235

**Published:** 2016-07-26

**Authors:** Junjia Wang, Reza Ashrafi, Rhys Adams, Ivan Glesk, Ivana Gasulla, José Capmany, Lawrence R. Chen

**Affiliations:** 1 Department of Electrical and Computer Engineering, McGill University, Montréal, Québec H3A 0E9, Canada; 2 Department of Physics, CEGEP Vanier College, Montréal, Québec H4L 3X9, Canada; 3 Department of Electronic and Electrical Engineering, University of Strathclyde, Glasgow, G1 1XU, UK; 4 ITEAM Research Institute, Universitat Politècnica de València, Valencia, 46022, Spain

## Abstract

An optical true time delay line (OTTDL) is a basic photonic building block that enables many microwave photonic and optical processing operations. The conventional design for an integrated OTTDL that is based on spatial diversity uses a length-variable waveguide array to create the optical time delays, which can introduce complexities in the integrated circuit design. Here we report the first ever demonstration of an integrated index-variable OTTDL that exploits spatial diversity in an equal length waveguide array. The approach uses subwavelength grating waveguides in silicon-on-insulator (SOI), which enables the realization of OTTDLs having a simple geometry and that occupy a compact chip area. Moreover, compared to conventional wavelength-variable delay lines with a few THz operation bandwidth, our index-variable OTTDL has an extremely broad operation bandwidth practically exceeding several tens of THz, which supports operation for various input optical signals with broad ranges of central wavelength and bandwidth.

Photonic signal processing offers fundamental solutions to overcome the bandwidth and processing speed limitations associated with electronic circuits. However, the small footprint and degree of integration for electronic circuits is arguably the most challenging issue to be achieved in photonic circuits. Similar to electronic processing circuits, to achieve a higher degree of integration in photonic processing circuits, the basic photonic building blocks need a fundamental innovation to be realized in a smaller footprint. An important basic building block is an optical delay line (ODL) that enables many photonic processing functions in optical communications and microwave photonic (MWP) systems. For example, ODLs are part of arrayed waveguide gratings (AWGs) for on-chip optical multi/demultiplexing functions in wavelength division multiplexed (WDM) systems^1^. ODLs have also been widely developed for microwave filtering and phase shift control devices that are ubiquitous in communications, radio-over-fiber, radar, radio astronomy, and digital satellite communication systems^2^,^3^, where very broad bandwidth radio frequency (RF) amplitude/phase filtering is necessary. In particular, MWP phase shift control based on ODLs is important for beam forming in phased array antennas^4^. There are a number of ODL features such as large total delay, small incremental delay steps for discretely tunable delays (e.g., for beam forming especially as the operating frequency of the phased array antenna increases) or continuous delay, and broad operating bandwidth. Not all of these features are required simultaneously and those that an ODL must provide will depend on the specific application. Among the different ODL implementations such as those based on all-fiber or micro-electro-mechanical-system (MEMS) technologies^5^,^6^,^7^, integrated waveguide ODL solutions are more compact and provide greater temporal resolution and correspondingly broader RF bandwidth.

The two general ODL approaches to induce a time delay (Δ*t*) between optical pulses (or signals) are based on (1) variation of the propagation group velocity (*v*_g_)^8^,^9^,^10^,^11^,^12^,^13^,^14^,^15^,^16^,^17^,^18^,^19^,^20^,^21^ and (2) variation of the propagation length (*L*)^22^,^23^,^24^,^25^, i.e., Δ*t* = Δ(*L*/*v*_g_), see Fig. 1(a,b). These two general approaches include the use of resonance enhancements whereby the physical length of the delay medium (e.g., waveguide) is enhanced through a cavity structure or by exploiting material resonances where the dispersion can be large. While continuously tunable ODLs based on resonance enhancements in cascaded microring resonator filters have been demonstrated^12^,^13^,^15^, there is a well-known trade-off between the amount of resonance enhancement that can be achieved and the operating bandwidth^26^. Here, we focus on ODLs that do not involve resonance enhancements. In the first approach illustrated in Fig. 1(a) 8,9,10,11,12,13,14,15,16,17,18,19,20,21, a highly dispersive waveguide is used to create group velocity variation along different wavelengths (λ). The pulses need to be carried on different optical wavelengths (or carrier frequencies), e.g., *λ*_1_, *λ*_2_, etc., to experience different propagation velocities and correspondingly, different propagation time delays. This is illustrated in the plot of *v*_g_ vs. *λ* in Fig. 1(d). This type of ODL has been implemented using photonic crystal waveguides^8^,^9^,^10^ and Bragg gratings^16^,^17^,^18^,^19^,^20^,^21^ and is normally referred to as a *wavelength-variable* ODL (i.e., it uses wavelength diversity). This optical time delay dimension of varying *v*_g_ along different wavelengths has applications in microwave photonic filtering schemes^8^,^9^,^10^,^11^,^12^,^13^,^14^,^15^,^16^,^17^,^18^,^19^,^20^,^21^; however, it cannot be used in many applications where the time delays must be obtained at the same wavelength, e.g., in AWGs^1^.

An ODL which provides time delays for pulses/signals with the ***same*** optical carrier/wavelength is one form of an optical true time delay line (OTTDL). The traditional implementation of such an OTTDL is depicted in Fig. 1(b) 22,23,24,25, where the differential/incremental time delay between the waveguides (also referred to as taps) is obtained by changing the waveguide length in each stage (i.e., the second ODL approach involving a *length-variable* design that exploits spatial diversity). For discretely tunable OTTDLs, the waveguides of different lengths are interconnected or cascaded via 2 × 2 or 1 × *N*/*N* × 1 switches (or splitters/combiners). To reduce the chip size, the waveguides are commonly arranged with spiral or curvy/serpentine topologies. For example, a 7-bit (discretely tunable) OTTDL with a maximum delay of 1.27 ns and 10 ps resolution (time delay increment) based on 8 cascaded 2 × 2 MZI switches and 7 waveguide paths in SOI with a total chip size of 7.4 mm × 1.6 mm was reported^25^.

One approach for minimizing or reducing complexities associated with the length-variable OTTDL is to develop an *index-variable* OTTDL where true time delay control can be realized through varying the group index/propagation velocity in the waveguides. Note that the ODL structure illustrated in Fig. 1(a) is not an OTTDL that provides time delays for signals at the same wavelength since the variable index/propagation velocity controls the relative wavelength-dependent delay and not the true time delay. Recently, a novel proposal of an index-variable OTTDL based on a heterogeneous multicore fiber has been introduced, where the true time delay of each tap can be controlled through designing a proper physical dimension and material doping concentration of each fiber core^27^,^28^. By tailoring an independent dispersion profile per core, a basic differential/incremental delay between taps of a few ps/km can be obtained. However, to date there has been no implementation of this concept to realize an integrated OTTDL.

In this work, we provide the first proposal and experimental demonstration of an *integrated index-variable* OTTDL in silicon-on-insulator (SOI). The device is based on subwavelength grating (SWG) waveguides, see Fig. 1(c), which allows for compact realizations that occupy minimal chip area. The structure has a straight waveguide array configuration in which the required incremental time delay between the waveguides/taps is achieved through a precise change of the group index/propagation velocity in each waveguide branch. Figure 1(d) illustrates the group velocity variation along the spatial dimension enabled by our approach as well as along the wavelength-variation dimension based on the wavelength-variable ODL method described above. In contrast to length-variable and wavelength-variable schemes, the optical delay generation process in our index-variable approach does not require any variation of waveguide length (*L*) and/or input pulse/signal wavelength (*λ*), as summarized in the table in Fig. 1(e). Our novel design provides a practical solution for achieving a small footprint and high degree of integration for this basic photonic building block, and accordingly increasing the performance of the photonic chip implementation of the above mentioned applications based on ODLs.

## Results

### Basic concept

Our integrated index-variable OTTDL is an innovative way of exploiting the already mature SWG waveguide technology. SWGs are microphotonic structures with high flexibility in terms of tailoring the effective index and dispersion^29^,^30^. An SWG waveguide is a grating structure with a period that is smaller than the wavelength of light, which suppresses the diffraction effects, and behaves as a homogeneous medium with an equivalent refractive index. The use of such SWGs in integrated photonics was first proposed in the 1990 s^31^. However, it has been with the more recent development of photonic technologies and high-resolution lithography that SWG structures have seen widespread application in integrated photonics in SOI. SWGs are normally classified into structures that are arranged crosswise or lengthwise relative to the light propagation direction. Crosswise SWG structures have been employed in various integrated photonic components such as fiber-chip grating couplers^32^, wavelength demultiplexers^33^, and devices incorporating high index-contrast structures^34^,^35^,^36^,^37^,^38^,^39^, such as high quality factor cavities^36^, hollow-core waveguides with very low propagation loss^37^, single-layer lenses and focusing reflectors with high focusing power^38^, and vertical-cavity surface-emitting lasers (VCSELs) with broadband operation range^39^. Lengthwise SWG structures have been employed for waveguiding^29^, waveguide crossings^40^, filtering based on ring resonators and Bragg gratings^41^,^42^, and ultra-broadband multimode interference (MMI) couplers^43^.

This work presents a revolutionary application landscape of lengthwise SWGs for implementing an integrated index-variable OTTDL and we demonstrate the concepts of our proposed solution using two different configurations. The first configuration is employed to induce a time delay between short optical pulses and the second configuration is used for microwave photonic phase shift control.

### First OTTDL configuration

**Figure 2:**
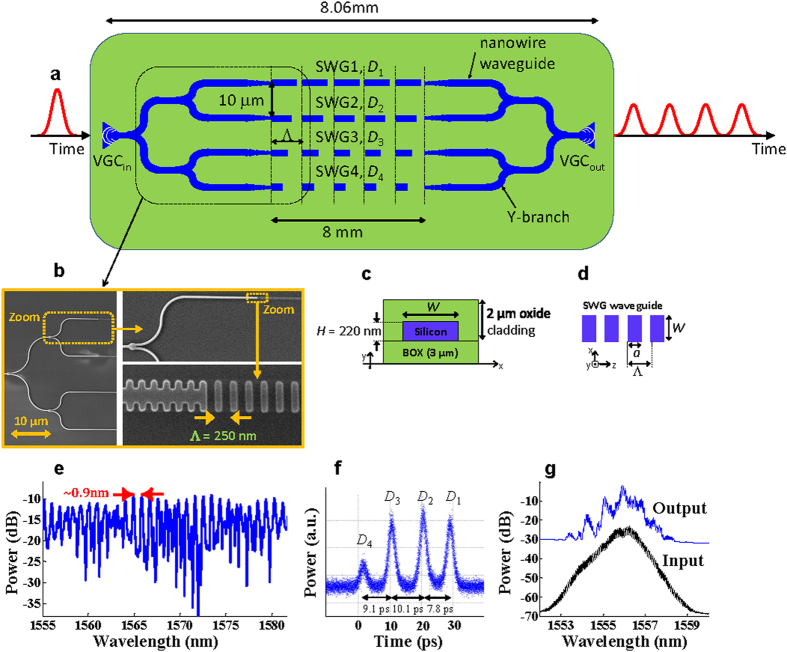
First OTTDL configuration. (**a**) Schematic and (**b**) a SEM image of our fabricated 4-tap OTTDL structure based on SWG waveguides in SOI. (**c**) The waveguide cross-section and (**d**) parameters of the SWG waveguides. (**e**) Measured power spectral response of the fabricated OTTDL. (**f**) Generated time-domain pulse train at the output of the fabricated OTTDL device in response to a single input optical pulse. (**g**) Input and amplified output signal spectra.

For the proof-of-principle demonstration of inducing time delays between optical pulses and for characterization of the SWG waveguide to synthesize different amounts of time delay, we have fabricated 4-arm and 2-arm Mach-Zehnder interferometers (MZIs), respectively. Figure 2(a) illustrates a schematic of the 4-arm MZI to implement a 4-tap OTTDL in SOI in a chip area of 0.24 mm^2^, i.e., 30 μm (width) ×8.06 mm (length). This index-variable OTTDL has been designed to have ~10 ps incremental time delay between the taps.

In our 4-tap OTTDL, each tap is based on one SWG waveguide, and all waveguides have the same length of *L* = 8 mm and are separated by 10 μm (based on the results in^44^, we do not expect any coupling or crosstalk between them). The SWG waveguides are realized by alternating periodically segments of silicon and silica, with a period of Λ = 250 nm along the propagation direction, see Fig. 2(d). By choosing the duty cycle *D* = *a*/Λ (where *a* is the length of the silicon segment in each period), the group index of the SWG waveguide can be modified and hence, the properties of the propagating mode (referred to as a Bloch mode) can be controlled^29^,^30^. The group index of the propagating Bloch mode in each tap is a function of *D* (see our obtained approximated equation in the Methods section). The corresponding generated incremental time delay between the taps can be expressed as


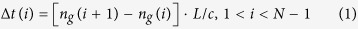


where *c* is the speed of light in vacuum and *N* = 4 is the number of taps in the OTTDL. By designing a different duty cycle value *D*_*i*_ for each respective SWG branch, different optical paths among the waveguides *all having the same length* (*L*) can be engineered to control the temporal separation between the taps. A cross-section of the SWG waveguide is shown in Fig. 2(c). The SWG waveguides are coupled to conventional silicon nanowire waveguides using a taper which converts a mode propagating in a silicon nanowire waveguide into a Bloch mode that propagates in the SWG waveguide^29^,^45^. A scanning electron microscope (SEM) image taken before the deposition of the top oxide cladding layer is shown in Fig. 2(b). The duty cycles in the SWG waveguides are *D*_1_ = 60%, *D*_2_ = 50%, *D*_3_ = 40%, and *D*_4_ = 30%. Six Y-branches with 50:50 splitting ratio and based on silicon nanowire waveguides^45^ are used to split and collect the optical power.

### Experimental results of the first configuration

Figure 2(e) shows the measured spectral response of the fabricated 4-tap OTTDL. The observed spectral periodicity features with ~0.9 nm bandwidth correspond to an incremental time delay step of ~9 ps between the taps. According to equation (1), this time delay difference between the taps corresponds to a group refractive index difference of 0.338 between the SWG waveguides. Also, the spectral features with smaller wavelength spacing correspond to the existing larger time delays in the OTTDL. Note that the shape and bandwidth of these features depend on the relative optical phase and propagation loss difference between the waveguides/taps. More accurate characterization of the time delay between the taps can be accomplished through the impulse response measurement or the time-domain measurement of the OTTDL response to a short optical input pulse. For this purpose, at the input of the OTTDL, we launched a 10 GHz train of Gaussian-like optical pulses with a full width at half maximum (FWHM) bandwidth of 2 nm at a central wavelength of 1556 nm. Figure 2(f) shows the generated time-domain output pulse sequence from a single input pulse measured using a fast optical sampling oscilloscope. As can be observed, the generated time delay differences between the taps of the OTTDL are 9.1 ps, 10.1 ps, and 7.8 ps, which are in general agreement with the spectral response measurements shown in Fig. 2(e). Note that, as shown in Fig. 2(f), the pulse propagating through the SWG waveguide with the lowest duty cycle (i.e., *D*_4_) arrives faster than the pulses in the other branches. The signal spectra at the input and output of the OTTDL are shown in Fig. 2(g). It is important to clarify the origin of the spectral shaping induced by the 4-tap OTTDL. The input signal is a 10 GHz pulse train and as such, there will be spectral lines separated by 10 GHz (~0.08 nm) in the corresponding optical spectrum [Fig. 2(g), black trace]. On the other hand, the output is a 4-bit sequence, i.e., a sequence of 4 pulses each spaced by ~9 ps, which repeats at 10 GHz. As a consequence, the spectrum of the output pulses will be different from that of the input. Moreover, the 4-tap OTTDL does not perform pulse repetition rate multiplication (i.e., from 10 GHz to 100 GHz) such that a ‘strong’ change in the output spectrum is expected.

The total fiber-to-fiber loss for an SWG waveguide depends on (1) coupling loss associated with the vertical grating couplers (VGCs) used for input and output coupling, (2) propagation loss in the SWG waveguide, and (3) loss due to mode mismatch between the nanowire waveguide and the SWG waveguide. The coupling loss is independent of the SWG waveguide duty cycle *D*. However, both the propagation loss and the loss due to mode mismatch are dependent on *D*, with the loss due to mode mismatch being more pronounced in SWG waveguides with smaller values of *D*^44^ (note that the loss due to mode mismatch is also polarization dependent though this is not an issue in our case since the VGCs are optimized for a single polarization only). While tapers reduce mode mismatch, a loss still exists; moreover, the taper should be optimized for each SWG waveguide duty cycle to minimize loss. In our proof-of-principle experiments, we used the same taper design for all 4 SWG waveguides for simplicity; as such, the total fiber-to-fiber loss between them is not the same and is highest for the SWG waveguide with *D*_4_ = 30%.

### Delay measurement from the spectral response

**Figure 3:**
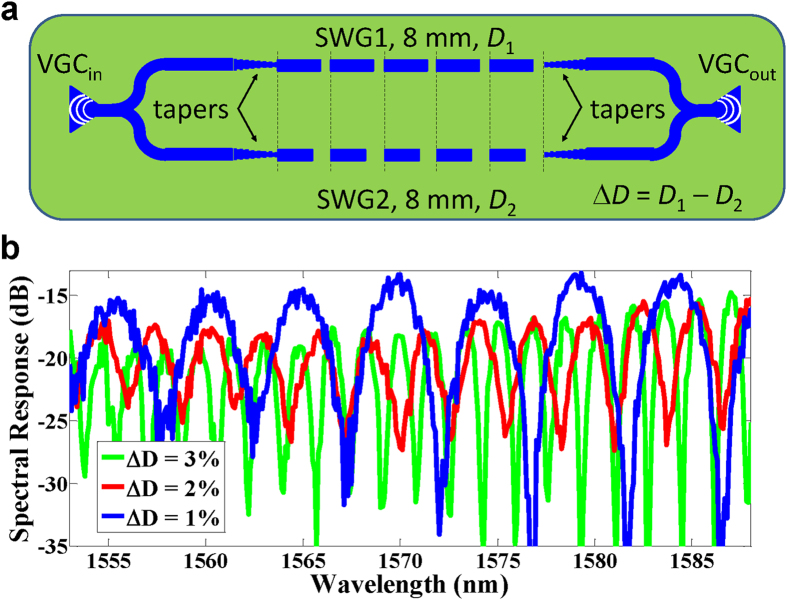
Investigating the delay increment based on spectral response measurements of a 2-arm MZI. (**a**) Schematic of our fabricated 2-arm MZI incorporating an SWG waveguide in each arm for direct time delay characterization from the spectral measurement. The difference between the duty cycle (Δ*D*) of the SWG waveguides in each arm determines the MZI FSR and the delay between the MZI arms. Three MZIs with Δ*D* = 1%, 2%, and 3% have been fabricated. (**b**) The measured SWG-based MZI spectral responses. The MZIs with Δ*D* = 1%, 2%, and 3% have FSRs of ~5 nm, ~2.9 nm, and ~1.7 nm, respectively, corresponding to the achieved delay difference of Δ*t* = 1.6 ps, 2.8 ps, and 4.7 ps between the MZI arms.

For direct time delay characterization from the spectral measurements, we have fabricated 2-arm MZIs incorporating SWG waveguides with different duty cycles in each arm, i.e., with a duty cycle difference of Δ*D*, see Fig. 3(a). By designing different values of Δ*D*, we can change/determine the MZI free spectral range (FSR) and correspondingly measure the time delay between the MZI arms. The SWG waveguides in each arm have the same length of 8 mm. We designed three MZIs with Δ*D* = 1%, 2%, and 3%. Figure 3(b) shows the corresponding measured spectral responses; the FSRs are ~5 nm, ~2.9 nm, and ~1.7 nm for MZIs with Δ*D* = 1%, 2%, and 3%, respectively, corresponding to a time delay difference of Δ*t* = 1.6 ps, 2.8 ps, and 4.7 ps between the MZI arms. The implemented SWG-based MZIs clearly show the potential of the proposed OTTDL solution for readily achieving small time delays of a few picoseconds in 8 mm long SWG waveguides. Note that the minimum achievable time delay scales down proportionally with the SWG length, and sub-picosecond time delay resolutions can be readily achieved in shorter waveguides (<8 mm).

### Second OTTDL configuration

**Figure 4:**
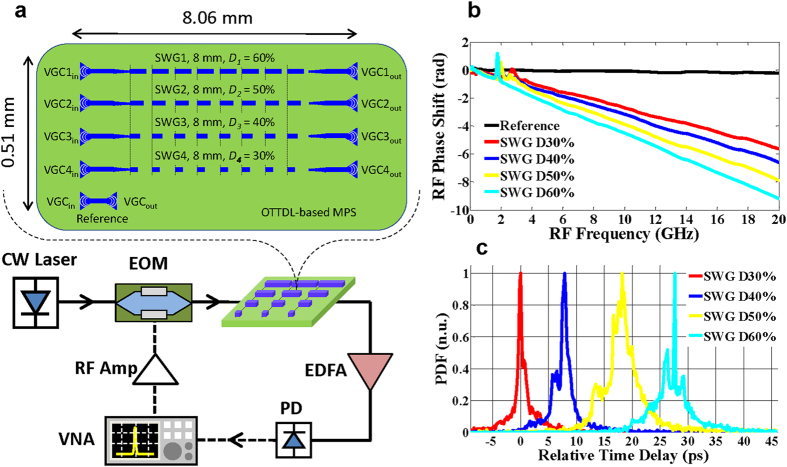
Second OTTDL configuration. (**a**) Experimental setup for measurement of the induced RF phase shift by our fabricated SWG-based OTTDL. (**b**) The measured RF phase shift when the modulated light is transmitted through different taps of the OTTDL with the duty cycle of SWG waveguide in each tap being *D*_1_ = 60%, *D*_2_ = 50%, *D*_3_ = 40%, and *D*_4_ = 30%. (**c**) The calculated probability distribution function (PDF) of the relative time delay given by the RF phase slope versus frequency shown in (**b**). The measured incremental time delay between the taps with *D*_1_ = 60%, *D*_2_ = 50%, *D*_3_ = 40%, and *D*_4_ = 30% are 8.9 ps, 10.7 ps, and 7.9 ps, respectively.

Figure 4(a) illustrates a schematic of our second OTTDL configuration which has been designed for photonic control of microwave phase shifting (MPS), i.e., OTTDL-based MPS. It is an array of 4 SWG waveguides of the same length (8 mm) and where the duty cycles are varied in 10% increments from *D*_1_ = 60% to *D*_4_ = 30%; a reference path comprising a short length of nanowire waveguide that connects the input and output VGCs is also included. The SWG waveguides are separated by 127.5 μm (as this separation is even greater than that used in the first OTTDL configuration, again, we do not expect any coupling or crosstalk between the waveguides). This OTTDL differs from the first configuration only in the sense that the input and output of the SWG waveguides are separate and not connected via the Y-branches. In this way, a microwave signal modulated on an optical carrier will experience a different phase shift when propagating through the different SWG waveguides in the OTTDL. The OTTDL occupies a chip area of ~4 mm^2^, i.e., 0.51 mm × 8.06 mm.

The measured RF phase shift as a function of frequency from the 4 different SWG waveguides is shown in Fig. 4(b). The time delay of each waveguide can be estimated from the average slope of the measured phase shift vs. frequency response. For this purpose, we have calculated the probability distribution function (PDF) of the slope of the phase shift (i.e., time delay) centered at different frequencies over the frequency range; the result is shown in Fig. 4(c). The mean values of the PDFs are then used to determine the relative time delays between the waveguides: the incremental time delays are 8.9 ps, 10.7 ps, and 7.9 ps between the SWG waveguides with *D*_1_ = 60%, *D*_2_ = 50%, *D*_3_ = 40%, and *D*_4_ = 30%, respectively, which are in good agreement with the delay measurements of 9.1 ps, 10.1 ps, and 7.8 ps between the taps from the first OTTDL configuration. As discussed in the Methods section, while the duty cycles of the SWG waveguides are varied in increments of 10%, this does not translate into a linear change in incremental time delay. In our proof-of-principle experiments, we used 10% increments for simplicity; the differences in duty cycles need to be chosen more carefully based on equations (1) and (5) in order to obtain a linear delay increment between taps. Note that the 20 GHz maximum RF frequency in our measurement setup is restricted by the bandwidth of the vector network analyzer (VNA) employed for the RF phase shift characterization.

## Discussion

The minimum/maximum incremental time delays and the total time delay that can be obtained depend in part on the minimum and maximum differences in duty cycle between the SWG waveguides that can be employed. At the same time, the total fiber-to-fiber loss for an SWG waveguide depends on the duty cycle. With 8 mm long SWG waveguides in SOI, a difference in duty cycle of Δ*D* = 1% results in an incremental time delay of 1.6 ps, which increases to 27.5 ps when Δ*D* = 30%. On the other hand, the variation in total fiber-to-fiber loss between SWG waveguides having a difference in duty cycle of Δ*D* = 30% is several dB. Thus, there is a balance between time delay and loss when selecting the value of Δ*D*.

The loss due to mode mismatch will be reduced when smaller differences in duty cycle are used. In this sense, using smaller values of Δ*D* may be preferred. We can compensate for the smaller changes in group index associated with smaller changes in Δ*D* by using longer waveguides to obtain a given incremental time delay. Our measured propagation losses in the SWG waveguides are ~2–3 dB/cm, which are similar to those in silicon nanowire waveguides. It is thus possible to use SWG waveguides of a few cm’s in length (e.g., as in^25^) to increase the incremental or total time delay. Note that if many taps are required in the OTTDL, smaller differences in duty cycle will be needed; this will help alleviate the variation in total fiber-to-fiber loss between the taps, even if the same taper design is used.

Reducing the incremental time delay can be achieved readily by reducing the length of the SWG waveguides so that sub-picosecond values are possible. Increasing the maximum incremental time delay (or the total time delay) will require an increase of the SWG waveguide length. To maintain a compact chip size, the SWG waveguides can be arranged in a serpentine manner. A 90° bending loss of ~1.5 dB was demonstrated in^44^ for a bend radius of 10 μm; this loss was reduced to ~1 dB when the bend radius was increased to 30 μm. These losses can be reduced even further by utilizing trapezoidal silicon segments (as opposed to rectangular silicon segments) in the SWG waveguide bends, as demonstrated in^46^.

In previously demonstrated wavelength-variable ODLs^8^,^9^,^10^,^11^,^12^,^13^,^14^,^15^,^16^,^17^,^18^,^19^,^20^,^21^, the maximum achievable time delay is restricted by the optical bandwidth of the device; in turn, this restriction defines the ultimate limit on the RF processing bandwidth, which is typically hundreds of GHz (note that this limit is regardless of bandwidth restrictions imposed by the instruments or components used in the measurement setup). On the other hand, the incremental delay between SWG waveguides can be maintained over large optical bandwidths such that the integrated index-variable OTTDL will not impose similar RF bandwidth limitations. In particular, the optical transmission window of SWG waveguides is constant over tens of THz (see further^29^,^30^); in practice, it is constrained largely by the bandwidth of the VGCs used for fiber coupling (the 3-dB bandwidth of our VGC couplers is ~25-30 nm or several THz). This will then support operation over broad RF bandwidths.

In our proof-of-principle experiments, we have focused on demonstrating an integrated index-variable OTTDL. To develop a (discretely) tunable OTTDL, we need a cascade of 2 × 2 switches and SWG waveguides of different duty cycles to obtain the incremental time delays per stage. In such tunable OTTDLs (see, e.g. refs 24 and 25), waveguides of different lengths must be used in each stage; however with our index-variable approach, SWG waveguides of the same length but different duty cycles are utilized. While SWG waveguide-based switches or modulators have yet to be demonstrated, the building blocks components exist for their implementation. On the other hand, it is possible to use conventional silicon nanowire waveguide components (Y-branches, MMI couplers, 2 × 2 MZI switches, etc.) and the SWG waveguides are exploited only for developing the index-variable delay elements.

Implementing a phased array radar system with a large number of elements (say *N*) requires the replication of multiple units of a basic delay line architecture^47^. As each delay unit is on chip, the overall system will benefit from a reduction in footprint with a compact delay structure. It should be noted that a passive 1 × *N* splitter is required for the replication, which induces a splitting loss above and beyond the total fiber-to-fiber losses discussed previously. As the number of taps/elements in the OTTDL increases, the splitting loss will increase. However, this is a fundamental limitation of any discrete time filtering scheme.

Our proposed approach provides a promising solution towards very-large-scale integration (VLSI) of OTTDL devices on photonic chips. Through the fairly straightforward SWG design strategy adopted, the taps of the OTTDL device can be fabricated in a compact array of straight waveguides with identical lengths. As with any OTTDL design, the SWG waveguides must be separated by a sufficient distance to avoid coupling and/or crosstalk. While the exact separation depends on duty cycle (stronger coupling has been observed for SWG waveguides with smaller values of *D*), separations of 10 μm will generally avoid coupling/crosstalk, which is small enough to ensure compactness.

## Conclusions

In summary, we have proposed and experimentally demonstrated a new concept for on-chip optical time delay realization without using waveguides with different lengths and/or curvy topology, by the precise control of the group index of the waveguides based on SWGs. In our approach, the group index and, consequently, the time delay of each OTTDL’s tap is controlled simply by changing the duty cycle of the SWG in the corresponding waveguide branch. This technique allows us to overcome critical limitations of present/conventional length-variable OTTDL methods, which can involve a complex device to generate optical time delays. Our demonstrated SWG-based OTTDL technique represents a practical and revolutionary solution for an ultra-compact implementation of this general purpose building block in photonic chips.

## Methods

### Integrated silicon SWG-based OTTDL fabrication

We fabricated the integrated SWG waveguide-based OTTDLs in SOI using electron beam lithography and a full etch at the University of Washington Nanofabrication Facility (WNF), a member of the NSF National Nanotechnology Infrastructure Network. The SWG waveguides have a cross-section of thickness *H* = 220 nm and a width *W* = 500 nm; they sit on top of a 3 μm BOX layer on a Si substrate and are covered by an index-matched cladding layer of thickness 2 μm. Each SWG waveguide has an input and output taper for coupling to a nanowire waveguide of the same cross-section. The tapers are based on a linearly chirped waveguide grating that is implemented using a uniform period of 200 nm and where the waveguide width is varied from 500 nm down to 200 nm over a length of 15 μm^45^.

### Group index and delay of SWG waveguides

The effective refractive index of SWG waveguides can be expressed as^30^:


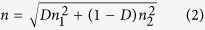


where *n*, *n*_1_, and *n*_2_ are the effective refractive indices of the SWG, silicon, and silica waveguides, respectively. The group index of the SWG waveguide (*n*_g_) can be calculated from the well-known definition of:


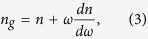


where *ω* is the optical angular frequency. By replacing equation (2) into equation (3), we obtain:


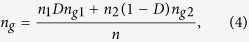


where the group index of the silicon and silica waveguides (*n*_g1_ and *n*_g2_, respectively) are defined according to equation (3).

By replacing equation (2) into equation (4), the group index of the SWG waveguide is obtained as:


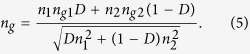


The simulated effective and group indices of silicon and silica for our waveguide dimension at the operation wavelength of ~1560 nm are estimated as: *n*_1_ = 2.4, *n*_g1_ = 4.4, *n*_2_ = 1.45 and *n*_g2_ = 1.47. According to equation (5), the group indices of the SWG waveguides with *D* = 60%, 50%, 40%, and 30% are obtained as *n*_g_ = 3.47, 3.2, 2.91, and 2.61, respectively. By using equation (1), the corresponding incremental time delays are estimated as 7.1 ps, 7.6 ps, and 8.2 ps, which are in a fairly good agreement with the experimental results from our fabricated OTTDL shown in Fig. 4(c). Note that from equation (5), the group index is not a linear function of duty cycle; as such, varying the duty cycle in a linear manner does not translate into a linear change in incremental time delay.

### Optical characterization

VGCs are used to couple light into and out of the devices (i.e., the two OTTDL configurations and the 2-arm MZI) and are optimized for TE transmission^45^. Two single-mode optical fibers are used to send and receive the input and output optical signals through the VGCs into and out of the chip, respectively. The optical spectral responses were measured by launching and sweeping a tunable laser source (Yenista Inc.) and capturing the output power using a power meter (ILX, FPM-8201). For the optical pulse source, we have used an actively mode-locked fiber laser (Pritel Inc.) with a repetition rate of 10 GHz. The output pulse train from the OTTDL was amplified using an erbium doped fiber amplifier (EDFA). The optical time trace of the pulse train at the output of the OTTDL, after amplification, was captured by a high-speed optical sampling oscilloscope with a bandwidth of 500 GHz (Alnair Labs). The spectrum of the input pulse and output pulse train from the OTTDL were captured using an optical spectrum analyzer (Agilent 86142B).

The total fiber-to-fiber loss of the first OTTDL configuration is ~32 dB (i.e., ratio of the output to input pulse energy), which includes the loss of the fiber-to-VGC coupling, each of which contributes about ~8 dB (this was measured using the reference path from the second OTTDL configuration), the splitting losses from the Y-branches, and the propagation loss through the SWG waveguides. In the second OTTDL configuration, the typical loss of a tap is 8 dB to 12 dB and includes the propagation loss as well as the loss due to mode mismatch/tapers; it is highest for the SWG waveguide with *D* = 30%).

### RF characterization

The RF phase response was measured by using a vector network analyzer (Agilent 8722ES) covering the RF frequency range from 0.2 GHz to 20 GHz. The experimental setup for the measurement of the RF phase shift introduced by our fabricated OTTDL is illustrated in Fig. 4(a). A continuous wave (CW) laser source operating at 1565 nm is modulated using an electro-optic modulator (EOM) driven by a VNA followed by a RF amplifier. The modulated light after propagating through the OTTDL, is amplified by an EDFA and then is detected by a fast photodiode (PD, HP 11982). The signal received by the PD is fed back to the VNA, which measures the phase of the transmitted signal. We use precision positioning stages for coupling to the different SWG waveguides for the phase measurements.

## Additional Information

**How to cite this article**: Wang, J. *et al*. Subwavelength grating enabled on-chip ultra-compact optical true time delay line. *Sci. Rep*. **6**, 30235; doi: 10.1038/srep30235 (2016).

## Figures and Tables

**Figure 1:**
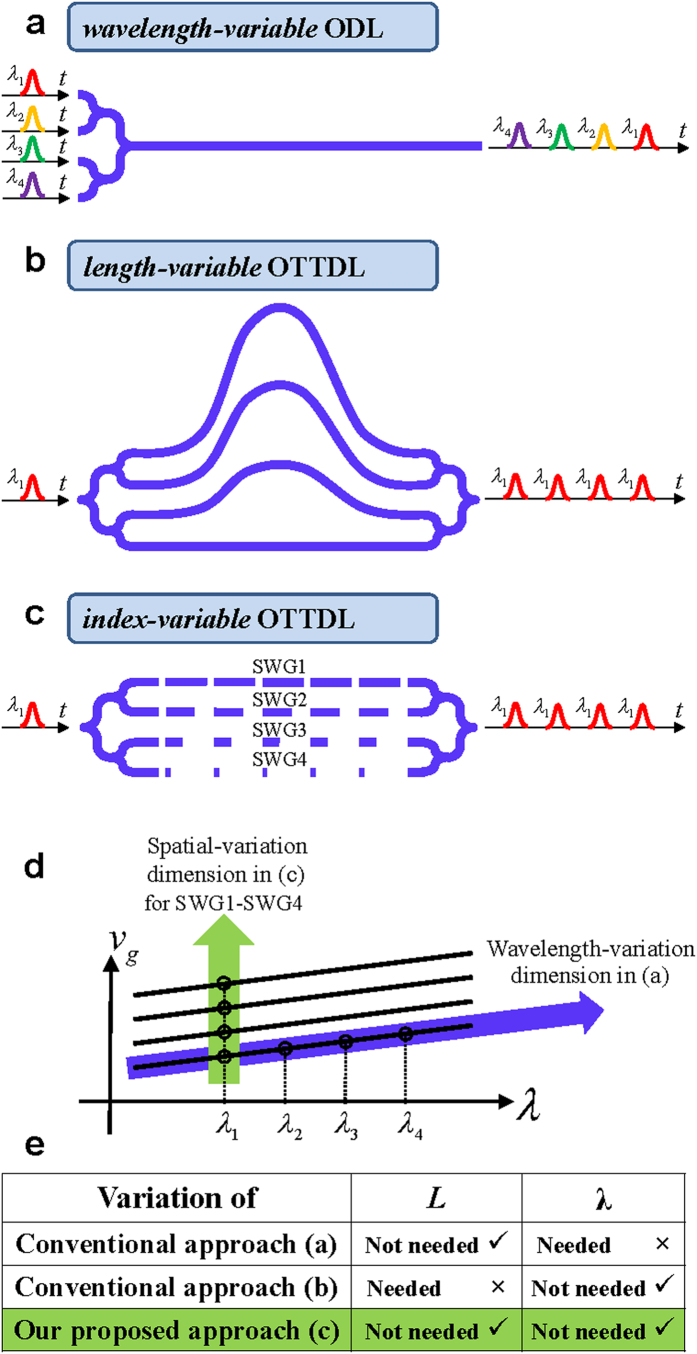
Illustration of the conventional approach for wavelength-variable ODL (**a**), the conventional approach for length-variable OTTDL (**b**), and our proposed approach of index-variable OTTDL (**c**). Two comparisons between these approaches are illustrated in the plot of *v*_g_ vs. *λ* in (**d**) and in the table in (**e**).
